# Investigation on the Properties of PMMA/Reactive Halloysite Nanocomposites Based on Halloysite with Double Bonds

**DOI:** 10.3390/polym10080919

**Published:** 2018-08-15

**Authors:** Shiwei Chen, Zhizhou Yang, Fuzhong Wang

**Affiliations:** Shandong Provincial Key Laboratory of Processing and Testing Technology of Glass & Functional Ceramics, School of Material Science and Engineering, Qilu University of Technology (Shandong Academy of Sciences), Jinan 250353, China; yzz000702@163.com (Z.Y.); wfz009@126.com (F.W.)

**Keywords:** polymer-matrix composites, thermal properties, mechanical properties, halloysite

## Abstract

PMMA/reactive halloysite nanocomposites were firstly prepared using reactive halloysite with double bonds. The halloysite was functionalized to improve its dispersion in the polymer matrix. The reactive halloysite could increase the molecular weight of PMMA. The molecular distribution of PMMA/reactive halloysite nanocomposite was more uniform than that of PMMA. The moisture absorption of PMMA/reactive halloysite nanocomposite increased with the addition of the reactive halloysite. Thermogravimetric analysis (TGA) and differential scanning calorimetry (DSC) confirmed that the thermal stability of PMMA/reactive halloysite nanocomposites was greatly enhanced. Significant improvement in the mechanical property of PMMA nanocomposites was achieved by the addition of 3 wt % reactive halloysite. A 31.1% increase in tensile strength and a 64.2% increase in Young’ modulus of the nanocomposites with 3 wt % of the reactive halloysite were achieved. Finally, the formation mechanism of PMMA/reactive halloysites nanocomposites was proposed. This approach demonstrated the potential for general applicability to other polymer nanocomposites.

## 1. Introduction

Nanocomposites are a new class of composite-filled particles, at least one dimension of which is in the range of nanometers [1,2,3,4]. There are three types of nanocomposites, depending on whether one, two, or three dimensions of the particles are in the range of nanometers [5,6,7,8]. Recently, polymer-layered silicate nanocomposites have attracted widespread interest for their improvement on the barrier properties, resistance to fire and ignition, and stiffness and toughness of the polymers [9,10,11]. Besides, the layered silicates used are easily available, cheap, and stable. Among the layered silicates, halloysite nanotubes (HNTs) are a special clay mineral with a hollow tubular structure. At present, it is a newly emerging nanomaterial esteemed for its biocompatible nature and environmental friendliness [12,13]. Stavitskaya reported that quantum dots (QD) were immobilized on the halloysite nanotubes’ surface to prepare the HNT–QD composite. HNT–QD systems were internalized by living cells and showed low cytotoxicity together with moderate photobleaching resistance. The halloysite clay composites are promising materials for bioimaging [14]. Fakhrullina investigated the toxicity of HNTs using the nematode Caenorhabditis elegans as a model organism. They found that halloysite is localised exclusively in the alimentary system and does not induce severe toxic effects on nematodes [15]. Makaremi prepared functional biofilms based on HNT/salicylic acid hybrids and pectin. Antimicrobial activity indicated the effective antimicrobial properties of pectin/halloysite-functionalized films and their potential to be used for food packaging applications [16]. Bugatti successfully prepared nanocomposites based on poly (*ɛ*-caprolactone) (PCL) and HNTs filled with lysozyme. It was demonstrated that it is possible to obtain controlled release systems for specific active packaging requirements with a simple mechanical treatment [17]. Bertolino reported the preparation and characterization of a nanocomposite based on HNTs and biopolymers. They opened new sustainable perspectives for the preparation of novel nanocomposites with layered structures that can be strategic for packaging, tissue engineering, and pharmaceutical applications [18,19]. Gorrasi reported new green biodegradable nanocomposites based on pectins and a nanohybrid composed of natural halloysite and rosemary oil. Antimicrobial activity indicated the potential application of the prepared nanocomposites in the packaging field [20]. The properties of the biocomposite films are affected by their mesoscopic structure. Du and Lvov reviewed that the thermal stability of polymer/HNT nanocomposites resulted from the hollow tubular structures of HNTs and, the barriers for heat and mass transport. The improved mechanical property of the polymers was correlated to the rod-like and high aspect ratio structures of HNTs and the unique surface chemical properties [21,22]. Cavallaro found that the mesoscopic structure, sizes, and polydispersity of HNTs were influenced by the specific geological origin of HNTs [23]. The good dispersion of halloysites and the interaction between polymers and HNTs play an important role in the enhancement of the property of the polymers. In order to achieve good dispersion, much research was done and many achievements were achieved in the area of nanocomposites [24]. Tang et al used phenylphosphonic acid to unfold the tubular structure of halloysite, which was then used to prepared epoxy–halloysite nanocomposites. The unfolded halloysites were effective additives to improve the fracture toughness of the cured epoxy without sacrificing other aspects of performance. The fracture toughness of the epoxy–halloysite nanocomposites was largely increased with an increase in the intercalation levels [25]. Qiu et al. reported thin membrane-like fully biodegradable nanocomposites, which were produced by blending individualized halloysite with polyvinyl alcohol (PVA). The halloysites were uniformly dispersed in PVA. Excellent mechanical properties of HNT–PVA nanocomposites were achieved. The DSC results indicated that HNT loading decreased glass transition temperature (*T*_g_) as well as crystallinity, while it increased the melt temperature (*T*_m_) of PVA [26]. Liu et al. prepared polymer/biodegradable and biocompatible clay nanocomposites via melt mixing. Halloysite was uniformly dispersed and oriented in the polylactide matrix. The modulus, toughness, and strength of the nanocomposites were largely higher than those of pure polylactide. Glass transition temperature and storage modulus of the nanocomposites also increased with the halloysite loading [27].

PMMA is used as a general material for its outstanding thermal stability, electrical performance, and optical clarity [28,29]. Recently, much more applications, both in industry and in academia, have placed higher demand on its performance, such as mechanical property and thermal stability. Incorporating nanoparticles into PMMA to form nanocomposites is an effective method to satisfy this requirement. Okolieocha prepared PMMA nanocomposites by incorporation of talc, carbon black (CB), and thermally reduced graphite oxide (TRGO), respectively. The fillers were well dispersed within the PMMA matrix, with the exception of CB. The inclusion of the respective fillers led to an increase of the tensile modulus compared to neat PMMA [30]. Li fabricated PMMA/multiwalled carbon nanotube (MWCNT) nanocomposites. They found that MWCNTs can further reduce the secondary neutron generation of the pure polymer. The enhanced thermal stability and the overall reinforcement of the polymer make MWCNT an effective filler for applications in the space industry [31]. Hammani prepared a PMMA/ZnO nanocomposite using solution mixing at the concentration range of 0–40 wt %. The improvement of the thermal and optical properties gives potential applications in different domains, i.e., electronics optics and photonics. Patra prepared PMMA nanocomposites filled with TiO_2_ nanorods. The good dispersion of TiO_2_ nanorods could effectively improve the thermal stability and thermomechanical properties [32,33]. Layered silicates have also been used to modify PMMA properties [34,35,36]. Li et al. prepared PMMA–kaolinite intercalation composites by the intercalation method. The incorporation of modified kaolinite into the polymer resulted in an increase in mechanical strength as well as thermal stability. The intercalated composites exhibited lower transparency and stronger resistance for UV spectra as kaolinite content increased [37]. Alexandro et al. investigated PMMA/organomodified montmorillonite (OMMT) nanocomposites. They found that the OMMT enhanced polymerization kinetics, especially in the gel-effect region, while sodium MMT acted rather as a reaction retarder. The OMMT increased the thermal stability, the glass transition temperature, and the average molecular weight of the nanocomposites. Their study displayed an increase in the Young’s modulus, with the amount of the OMMT accompanied by a decrease in the elongation at break and tensile strength [38]. However, to our best knowledge, there has no study yet been conducted on preparing PMMA nanocomposite films using halloysite. The main reason was that the weak polarity of methyl methacrylate made it difficult for halloysite to disperse well in the polymer matrix, which had an adverse effect on the performance of PMMA.

Here, we obtained reactive halloysite with double bonds, which was then used to prepare PMMA/reactive halloysite nanocomposites. The novelties of the research were: this is the first instance of preparation of the reactive HNTs with double bonds with the assistance of both tetraethoxysilane and silane agent, with systematic investigation of the effect of this reactive halloysite on the properties of PMMA nanocomposites.

## 2. Experiment

### 2.1. Materials

Halloysite nanotubes (HNTs) were supplied by SanXing High-New Material Company of Zaozhuang, China. The length of the HNTs was in the range of 300 nm–2.2 μm, with the outer diameter of 40–90 nm and the inner diameter of 10–15 nm. Tetraethoxysilane (TEOS) and 3-methacryloxypropyltrimethoxysilane (KH570) were purchased from Beijing Chemical Reagents Company, Beijing, China. TEOS and KH570 were of commercial grade. Methyl methacrylate (MMA) from Beijing Chemical Reagents Company (Beijing, China) was of commercial grade and used as distilled. Potassium persulfate, ammonia (28 mass %), sodium dodecylsulfate (SDS), and anhydrous ethanol were of analytical grade and supplied from Tianjing Damao Chemical Reagent Factory, Tianjin, China. Deionized water was used in all experiments.

### 2.2. Functionalization of Reactive HNTs

Firstly, the desired amount of dried HNTs, ammonia (18.4 mL), and ethanol (100.0 mL) were added in the flask with stirring for 2 h at room temperature. When the temperature was heated to 60 °C, TEOS (2.0 mL) was put in. The mixture was stirring for 6 h. The slurry was obtained after the filtration and washing with ethanol several times. The product was dried at 110 °C for 12 h, grinded, and sieved through a 250 µm mesh. The product yield was 91%. The obtained product was named T-HNTs.

Secondly, the desired amount of T-HNTs was dispersed in the ethanol (100.0 mL) again, followed by magnetic stirring for 2 h. Meanwhile, KH570 (2.0 mL) was added dropwise into the mixture of ethanol (10.2 mL) and water (0.8 mL). After stirring for 1 h, the pH of the mixture was adjusted to 4–5 by acetic anhydride to obtain the hydrolyzed KH570. Then, the hydrolyzed KH570 was added into the slurry above, followed by stirring for 2 h. The slurry was filtrated and washed with ethanol several times. The product was then vacuum-dried at 110 °C for 12 h, grinded, and sieved through a 250 µm mesh. The obtained product was the reactive HNTs, termed as KT-HNTs. The yield was 92%.

In order to make a comparison, HNTs were modified with KH570 directly and the product was termed as K-HNTs. The yield was 84%. The pristine HNTs were termed as P-HNTs.

### 2.3. Preparation of PMMA/Reactive Halloysite Nanocomposites

PMMA/reactive halloysite nanocomposite was prepared via emulsion polymerization with potassium persulfate as the initiator and SDS as emulsifier. The desired amount of KT-HNTs, SDS (0.2 g), MMA (21.4 mL), and deionized water (90.0 mL) were put in a 250 mL three-necked round flask. The solution was kept stirring for 30 min at room temperature. Then, nitrogen was purged into the flask for another 30 min. When the temperature was up to 75 °C, 0.9 wt % potassium persulfate solution (10 mL) was added dropwise into the flask. Five hours later, the reaction was done. The product was filtered, washed with ethanol, and vacuum-dried at 110 °C for 12 h. The yield was about 90%. The product with 3 wt % of KT-HNTs was termed as PMMA-KT-3%.

To make the comparation, the products with 3 wt % of pristine HNTs and T-HNTs were prepared according to the process above and were termed as PMMA-H-3% and PMMA-T-3%, respectively. Pure PMMA was also prepared via emulsion polymerization.

### 2.4. Preparation of PMMA/Reactive Halloysite Nanocomposite Thin Films

PMMA/reactive halloysite nanocomposite (1.0 g) was dissolved in 1-methyl-2-pyrrolidinone (9.5 g) with continuous stirring for 24 h at room temperature. The solution was put onto a clean glass plate and evaporated at 80 °C for 24 h to obtain the films. The thickness of these films was about 0.08 mm.

### 2.5. Characterization

The X-ray diffraction of the sample was measured on a Siemens D-500 diffractometer (Madison, WI, USA). The operation was conducted under the condition of 40 kV and 30 mA, using filtered Cu Kα radiation. The angle 2θ was varied from 3° to 60°.

The measurement of Fourier Transform infrared spectroscopy (FTIR) was performed on a Spectrum 1000 Perkin-Elmer spectrometer (Germany)in the spectral area of 400–4000 cm^−1^ with a resolution of 4 cm^−1^ and 32 scans.

The measurement of the thermal stability for the samples was operated using a Thermogravimetric Analysis and Differential Thermal Analysis (TG-DTA) apparatus (Mettler-Toledo GmbH, Greifensee, Switzerland). The condition was under a nitrogen flow of 25 cm^3^ min^−1^ for the sample and 10 cm^3^ min^−1^ for the balance, with a heating rate of 20 °C min^-1^ from 25 °C to 1000 °C. Differential thermal gravity (DTG) curves were the first-order derivative curves of mass to time.

The microstructures of the samples were measured by a FEI XL 30 scanning electron microscope (FEI Company, Eindhoven, Netherlands). The voltage of the electron beam used for SEM was 20 kV. The specimens were coated with a thin carbon film prior to SEM observations.

Differential Scanning Calorimetry (DSC) (TA Instruments, New Castle, DE, USA) was used to measure the glass transition temperatures of the samples. The glass transition temperatures were acquired by heating up to 180 °C with a rate of 20 °C/min.

The average molecular weights and the molecular weight distribution of the samples were determined by Gel Permeation Chromatography (GPC) (model PL-GPC 50, England). The elution solvent was tetrahydrofuran with a constant flow rate of 1 mL/min.

UV–visible transmission spectra were measured according to ASTM D 1003 and performed on a Cary-100 UV–visible spectrophotometer (Santa Clara, CA, USA). The results were acquired as average of the values from three samples. The attenuation coefficient (k) for each sample was computed as

k = A/(2.3D)(1)

where A is the absorbance and D is the thickness of the rectangular film as measured with a micrometer (± 10^−3^ mm).

Moisture absorption tests were performed according to the standard ISO 62:2008. Specimens were made as squares of 5 × 5 cm^2^. At room temperature, specimens were immersed in distilled water for different hours, wiped dry, and immediately weighed. Water absorption was obtained by the increase in specimen weight divided by the original weight.

The mechanical properties of the samples were performed on an Instron testing machine Model 55 67 (Bensenville, Illinois, United States) according to ASTM D638. Tests were operated at 1 kN load cell with a crosshead speed of 2 mm/min. Five specimens for each sample were used for measurement and the result was calculated as average of the values.

## 3. Results and Discussion

### 3.1. Characterization of the Reactive Halloysites

**Figure 1:**
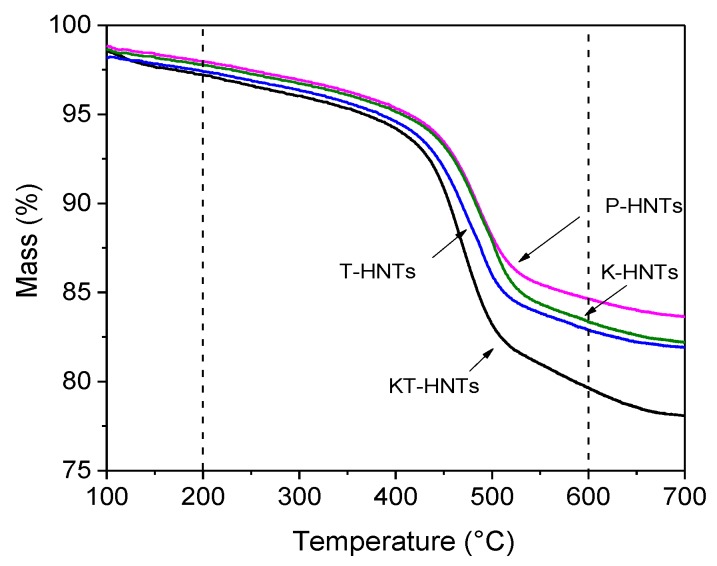
TGA curves of P-HNTs, K-HNTs, T-HNTs, and KT-HNTs.

Figure 1 displayed the TGA curves of P-HNTs, K-HNTs, T-HNTs, and KT-HNTs. As reported in the article [39], TEOS and the coupling agent degrade when the temperature is within the range of 200 °C and 600 °C. The difference in the weight loss of P-HNTs and T-HNTs within this range was corresponding to the amount of TEOS on the T-HNTs. The amount of TEOS on T-HNTs was about 2.2 wt %. The difference on the weight loss of P-HNTs and K-HNTs within this range was corresponding to the amount of coupling agent on the K-HNTs. Similarly, the difference on the weight loss between KT-HNTs and T-HNTs was ascribed to the degradation of the coupling agent. According to Figure 1, the amount of the coupling agent on the K-HNTs was about 1.0 wt %, less than the 3.2 wt % on the KT-HNTs, indicating that coupling agent modified the halloysite and TEOS was beneficial to modify the halloysite.

**Figure 2:**
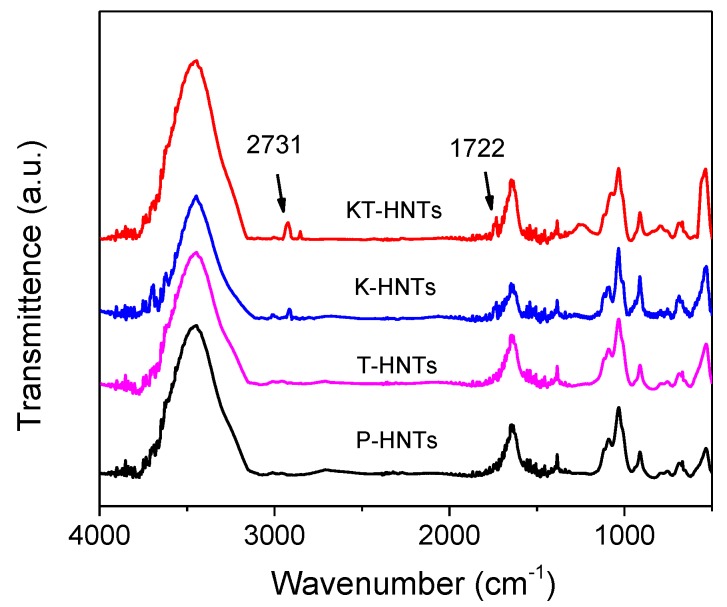
FTIR spectra of pristine HNTs and modified HNTs, P-HNTs, T-HNTs, K-HNTs, and KT-HNTs.

Figure 2 displayed the FTIR spectra of (a) HNTs, (b) T-HNTs, (c) K-HNTs, and (d) KT-HNTs. Compared to the unmodified P-HNTs, K-HNTs and KT-HNTs exhibited two new peaks, at 2931 cm^−1^ and 1722 cm^−1^, which were ascribed to the C–H stretching vibration bands and C=O symmetrical stretching vibrations. This indicated the silane agent grafted on the surface of HNTs. It should be noted that the intensity of the new peaks of KT-HNTs was stronger than that of K-HNTs, indicating that more silane agent was grafted on K-HNTs. The results showed that TEOS was beneficial for the silane agent to be grafted on HNTs.

Halloysite is a natural hydrated polymorph of kaolinite, with a spacing (d_001_) of 1 nm. The space layer of HNTs is filled with a monolayer of water molecules, which are weakly held in the space layer. So, HNTs could be dehydrated and transformed to the halloysite with a spacing of 0.73 nm [40].

**Figure 3:**
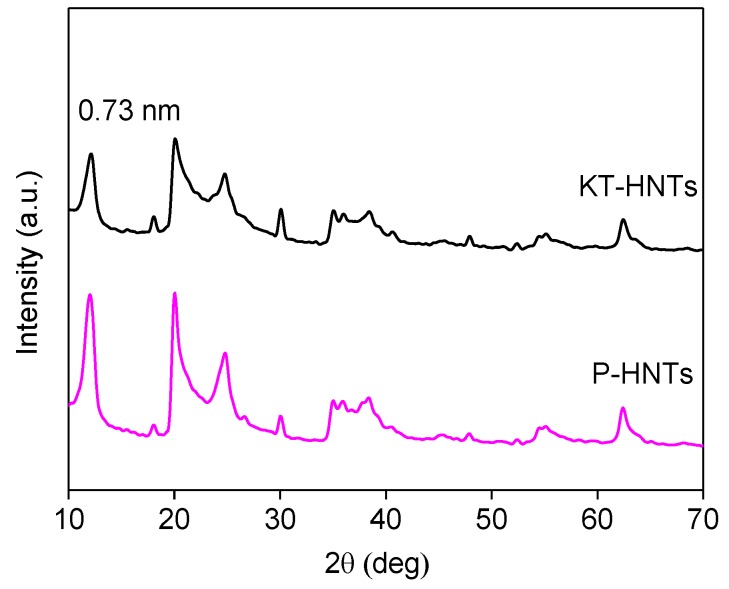
XRD diffraction patterns of P-HNTs and KT-HNTs.

XRD was used to measure the change of the crystal structure of pristine halloysites and reactive halloysites. As illustrated in Figure 3, the typical diffraction peak (2θ = 12.1° and 20.0°) of halloysite occurred in the XRD curves of KT-HNTs. According to the Bragg equation, the corresponding layer distance of KT-HNTs was 0.73 nm, which was equivalent to that of P-HNTs. The result confirmed that the TEOS and KH570 did not intercalate with the layers of halloysites. It should be noted that the peak intensity of KT-HNTs at 2θ = 12.1° and 20.0° was weaker than that of P-HNTs, showing that the modifying agents, TEOS and KH570, had an impact on the peak intensity of halloysites.

**Figure 4:**
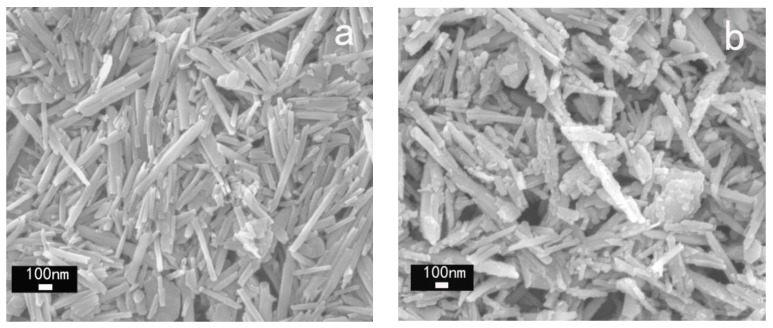
SEM images of (**a**) P-HNTs and (**b**) KT-HNTs.

Figure 4 displayed SEM images of P-HNTs and KT-HNTs. It is found that the natural P-HNTs are smooth, clean, and uniform tubes. The surface of the halloysite had a distinct outline, while the surface of KT-HNTs was rough and the outline was indistinct, which was obviously different from the morphology of P-HNTs.

### 3.2. Characterization of PMMA/Reactive Halloysite Nanocomposites

**Figure 5:**
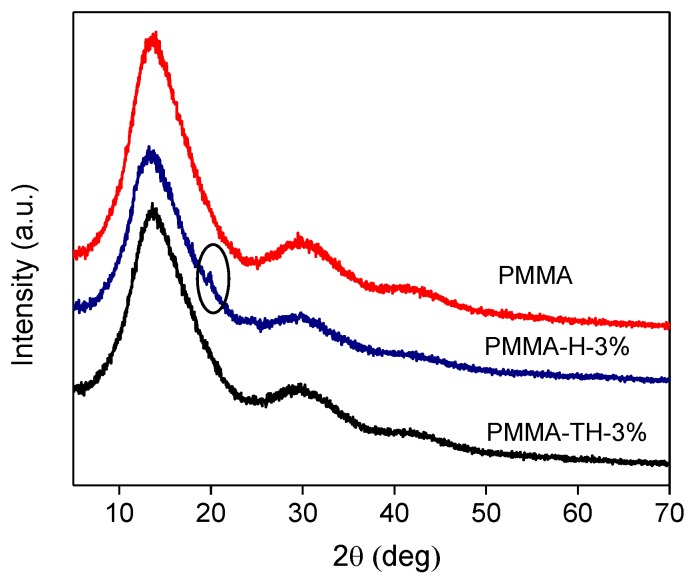
XRD diffraction patterns of PMMA and PMMA/reactive halloysite nanocomposite.

The XRD diffraction patterns of PMMA and PMMA/reactive halloysite nanocomposite were illustrated as Figure 5. It is seen that the wide diffraction peak occurred in the XRD diffraction patterns of PMMA, indicating the structure of PMMA was amorphous. Meanwhile, the typical diffraction peak (2θ = 20.0°) of the halloysite occurred in the XRD diffraction patterns of PMMA-H-3%, whereas this peak disappeared in the XRD diffraction patterns of PMMA-TH-3%, which was probably ascribed to better compatibility and stronger interaction between PMMA and reactive HNTs.

Average molecular weights and polydispersity of the molecular weight distribution of PMMA and PMMA/reactive halloysite nanocomposite were illustrated as Table 1. It is found that the weight-average molecular weight (*M*_w_) and number-average molecular weight (*M*_n_) of PMMA nanocomposite were higher than those of pure PMMA. It is reasonable that the mechanism to prepare PMMA was a free radical reaction, which was obviously affected by diffusion control. The free radicals from the initiator could initiate the reaction, react with the monomer, and thus prolong the polymer chain. Meanwhile, large free radicals, which could come across each other and terminate the reaction, would occur during the process. The halloysite could restrict the motion of large free radicals and reduce the possibility of their meeting, thus resulting in that the large free radicals remained to react with monomers. It should be noted that the molecular weight of PMMA-TH-3% was larger and the *M*_w_/*M*_n_ value was lower than that of PMMA-H-3%. This was because the reactive groups on the KT-HNTs could have strong interactions with the polymer matrix, which would prevent further the motion of large free radicals. In addition, *M*_w_/*M*_n_ of PMMA-TH-3% was lower than that of PMMA, showing that the weight distribution of PMMA nanocomposites was more uniform.

**Figure 6:**
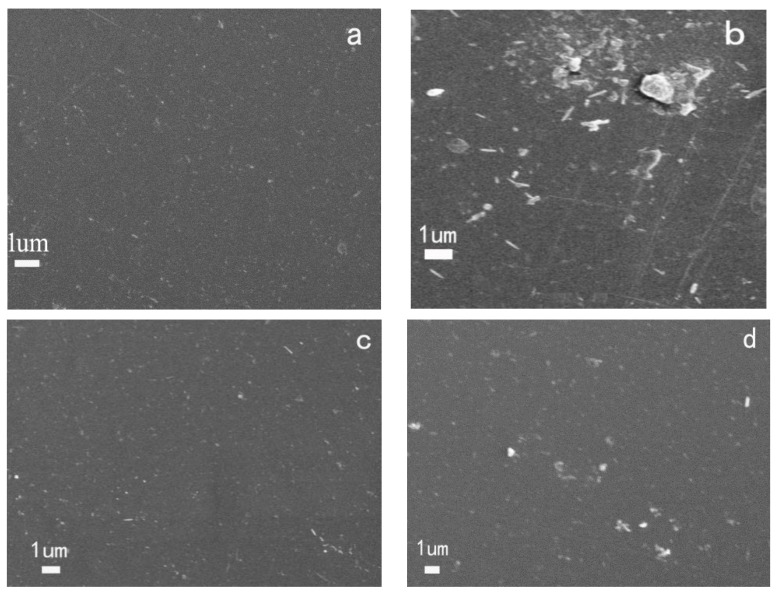
SEM images of (**a**) PMMA-TH-1%, (**b**) PMMA-H-3%, (**c**) PMMA-TH-3%, and (**d**) PMMA-TH-5%.

The SEM images of PMMA-TH-1%, PMMA-H-3%, PMMA-TH-3%, and PMMA-TH-5% are displayed in Figure 6. It can be seen that large particles occurred in the PMMA-H-3%, indicating the pristine halloysites aggregated easily, whereas there are nearly no large particles in the PMMA-TH-3%, showing that KT-HNTs could be dispersed averagely and KT-HNTs had good dispersion in the polymer matrix. Meanwhile, when the content of KT-HNTs was 5 wt %, some large particles occurred in the SEM images.

**Figure 7:**
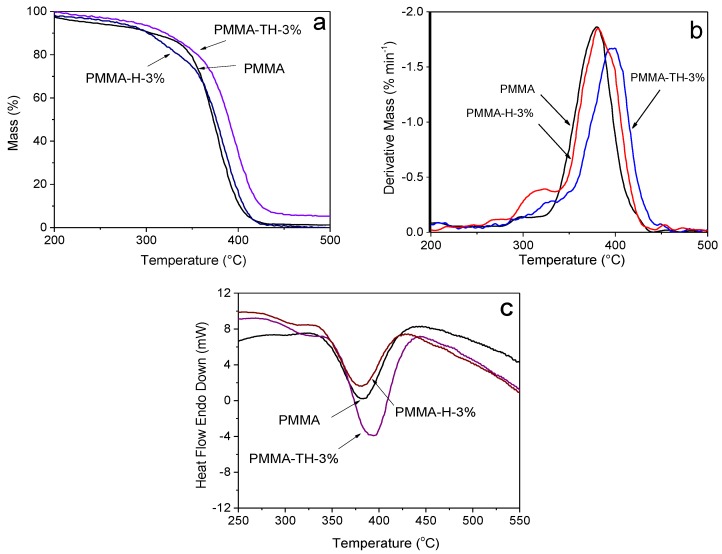
(**a**) TGA, (**b**) DTG, and (**c**) DTA curves of PMMA and PMMA/reactive halloysite nanocomposite.

Figure 7 displayed the TGA, DTG, and DTA curves of PMMA and PMMA/reactive halloysite nanocomposite. The degradation temperatures at 5 wt % and 10 wt % loss are illustrated in Table 2. It is found that the beginning degradation temperature of PMMA was nearly equivalent to that of PMMA/reactive halloysite nanocomposite. Compared to pure PMMA, the temperatures at 5 wt % loss and 10 wt % of PMMA-TH-3% increased by 46 °C and 25 °C, respectively. Meanwhile, DTG curves showed that the peak position of PMMA-TH-3% shifted toward a higher temperature. These results showed an increased thermal stability in the nanocomposites, indicating that KT-HNTs were beneficial to improving the thermal stability of PMMA. The reasoning was as follows: firstly, halloysite had good thermal stability; secondly, halloysite had good barrier function, which could restrict the energy transformation; thirdly, reactive groups on KT-HNTs could interact with the polymer matrix and form the network, further preventing the escape of small molecules. In addition, the entrapment of the volatile products of the degraded polymer within the HNT cavity was beneficial to enhance the thermal stability of PMMA nanocomposites [16]. The temperature at 10 wt % loss of PMMA-H-3% decreased by 4 °C compared to pure PMMA, which was ascribed to the large aggregates and phase separation between polymer and filler observed in SEM images.

As displayed in Figure 7c, the peak area and peak temperature of PMMA-TH-3% were larger than those of PMMA and PMMA-H-3%, confirming that PMMA-TH-3% had greater thermal stability and KT-HNTs were more beneficial to improve the thermal stability of PMMA, which was identical to the result of TGA above.

**Figure 8:**
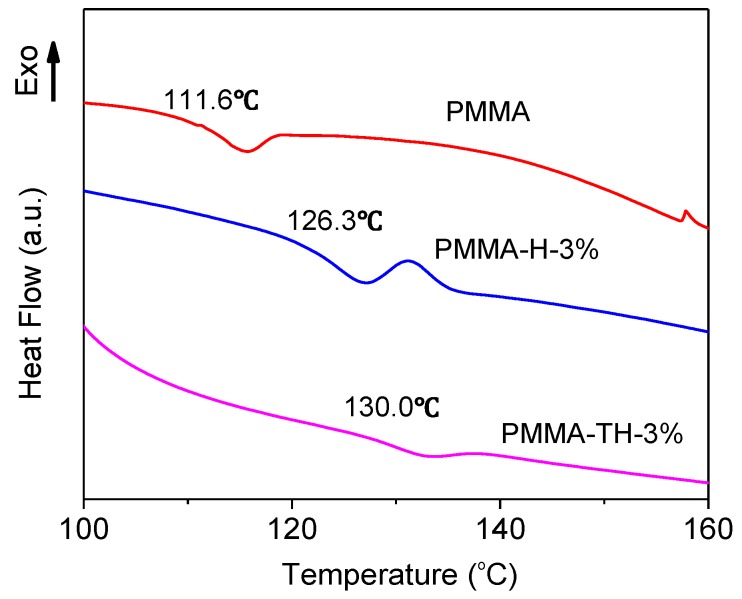
DSC curves of PMMA and PMMA/reactive halloysite nanocomposite.

DSC curves of PMMA and PMMA/reactive halloysite nanocomposite are displayed in Figure 8. It is found that the glass transition temperatures of PMMA-H-3% and PMMA-TH-3% increased compared to PMMA. It was reasonable that KT-HNTs and P-HNTs could restrict the motion of the chain. Meanwhile, PMMA-TH-3% had a higher glass transition temperature than PMMA-H-3%. This was because KT-HNTs could have strong interaction with the polymer matrix and even react with the polymer, thus preventing further the motion of the chain.

The molecular weight was another factor influencing the glass transition temperature. The larger molecular weight could further restrict the chain motion. According to the results of GPC, PMMA-TH-3% had a larger molecular weight than PMMA-H-3%, which was exactly corresponding to the result of DSC.

**Figure 9:**
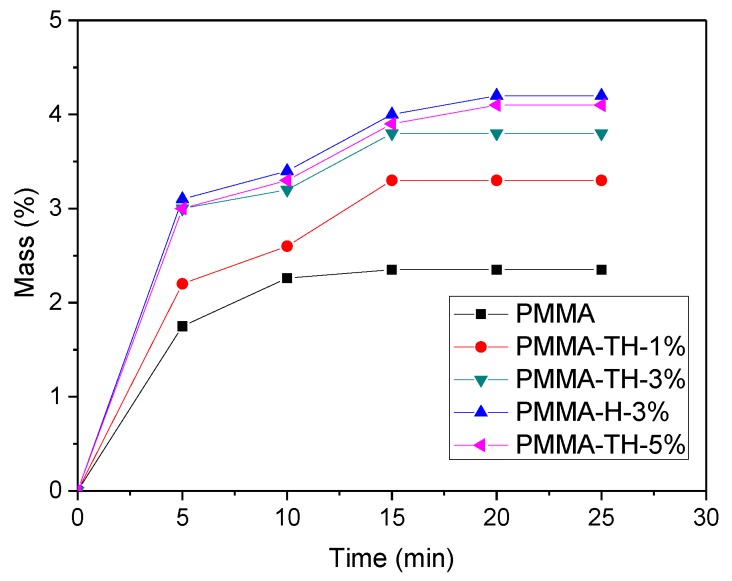
Moisture absorption curves of PMMA/reactive halloysite nanocomposite films.

Figure 9 shows the moisture absorption curves of the PMMA/reactive halloysite nanocomposite films. It was seen that the moisture absorption of the PMMA/reactive halloysite nanocomposite films was greater than that of PMMA. Compared to PMMA-H-3%, the moisture absorption of the PMMA/reactive HNTs nanocomposite films was less. The reasoning was as follows: firstly, KT-HNTs had good compatibility with the polymer matrix, and the interface between KT-HNTs and the polymer matrix was more compact than that between P-HNTs and the polymer matrix; secondly, pristine halloysite easily aggregated to large particles, resulting in defect interfaces. The interfaces could store up water.

In addition, moisture absorption of the PMMA nanocomposite films increased with the content of KT-HNTs. It is reasonable that more KT-HNTs would easily aggregate to large particles, resulting in more defect interfaces and increasing the moisture absorption.

**Figure 10:**
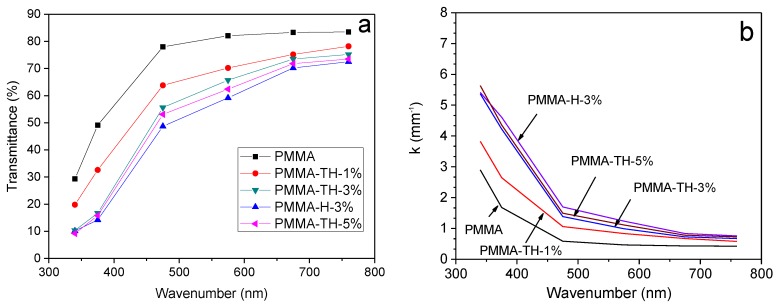
(**a**) Light transmittance and (**b**) attenuation coefficient of PMMA/reactive halloysite nanocomposite films.

Light transmittance of the PMMA/reactive halloysite nanocomposite films is illustrated in Figure 10a. It is found that KT-HNTs decreased the light transmittance of films and the transmittance decreased with the addition of KT-HNTs. This was because the light could be scattered by the halloysite. As the content of KT-HNTs increased, more aggregates occurred, resulting in lower light transmittance. Moreover, the light transmittance of PMMA-H-3% was worse than that of PMMA-TH-3%. It was reasonable that the reactive halloysite had better dispersion than pristine halloysite in the polymer matrix.

The dependence of the attenuation coefficient (*k*) on (*λ*) for the PMMA/HNT nanocomposites are shown in Figure 10b. According to Figure 10b, for all of the samples, one observes a larger k value at lower *λ*. The *k* versus *λ* trends toward higher values with the increase of the HNTs in the PMMA nanocomposites. Meanwhile, the k value of PMMA-TH-3% was lower than that of PMMA-H-3%. The phenomenon is certainly influenced by the filler concentration, the morphology of the filler, and the tendency to form aggregates [41]. The result is in a very good agreement with the microscopic structure evidenced by SEM experiments.

The mechanical property of PMMA/reactive halloysite nanocomposite films is listed in Table 3. It was found that KT-HNTs improved the mechanical property of PMMA. When the content of KT-HNTs was less than 3 wt %, the tensile strength of nanocomposite film increased with the amount of KT-HNTs. The tensile strength of PMMA-TH-3% could be up to 42.1 MPa, which was larger than that of PMMA (32.1 MPa). The reasoning was as follows: firstly, KT-HNTs were dispersed averagely in the polymer matrix, which could reduce the stress concentration and sustain much stress; secondly, good compatibility and strong interaction between KT-HNTs and the polymer matrix could further improve the tensile strength. At the same time, the tensile strength of PMMA-TH-3% was larger than that of PMMA-H-3%, indicating that reactive halloysite was more beneficial to enhance the tensile strength. However, when the content was too high, such as 5 wt %, the tensile strength of PMMA nanocomposites decreased.

Compared to pure PMMA, the elongation at break of PMMA nanocomposites decreased. This was ascribed to weak interface interaction between KT-HNTs and the polymer matrix, which could not sustain the distortion. Meanwhile, the elongation at break of PMMA-TH-3% was higher than that of PMMA-H-3%. This was because the interaction between KT-HNTs and the polymer matrix was stronger than that between P-HNTs and the polymer matrix. When the content of KT-HNTs was 5 wt %, the elongation at break of PMMA-TH-3% decreased. The reason was as follows: more particles were not dispersed well in the polymer matrix, resulting in more defect interfaces. The defect interfaces could become many cavities, which could be easily broken. 

In addition, the Young’s modulus of PMMA nanocomposite films increased firstly and then decreased as the content of KT-HNTs increased. When the content of KT-HNTs was 3 wt %, the Young’s modulus of the PMMA nanocomposites had the largest value. The reason was as follows: the interaction between KT-HNTs and the polymer matrix got stronger, leading to a more stable structure and larger stiffness of the film. However, more particles would aggregate and reduce the structural stability of the films when the content was 5 wt %. Finally, the Young’s modulus of the film decreased.

It should be noted that the tensile strength and Young’s modulus of PMMA-TH-3% were larger than that of PMMA-KH-3%, showing that the HNTs modified by both TEOS and coupling agent were more beneficial to enhance the strength of PMMA than HNTs modified only by coupling agent.

### 3.3. The Formation Scheme of PMMA/Reactive Halloysite Nanocomposites

**Figure 11:**
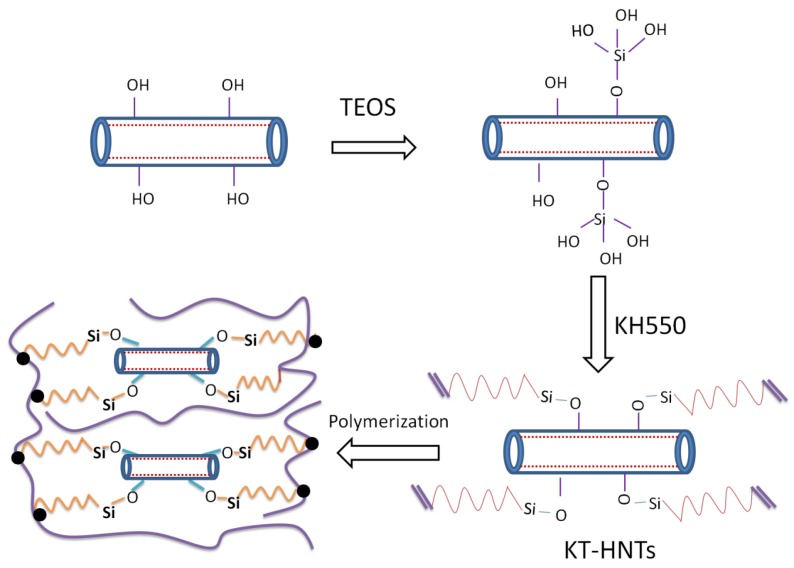
The formation mechanism of PMMA/reactive halloysite nanocomposite.

Based on the results of XRD, SEM, and the properties of PMMA nanocomposites, the formation mechanism of PMMA nanocomposites was proposed as illustrated in Figure 11. More hydroxyls occurred on the surface of halloysite modified by TEOS. Then, the coupling agent could easily react with the hydroxyls, and thus introduce more double bonds to the surface of the halloysite, which was more beneficial than direct modification with coupling agent. The obtained halloysite had good dispersion in the polymer matrix and was compatible with the polymer. The strong interaction and even the chemical reaction between the reactive halloysite and polymer matrix led to the formation of the PMMA nanocomposites with excellent properties.

The reactive halloysite could restrict the motion of large free radicals, reducing the possibility of their meeting, and thus the reaction would go on (large free radicals could react with each other and terminate the reaction). The reactive halloysite could absorb the heat, prevent its transformation, and restrict small molecules escaping from the nanocomposites, leading to the improvement of the thermal stability. Meanwhile, the reactive halloysite could restrict the motion of the polymer chain and thus increase the glass transition temperature. In addition, the good compatibility between the reactive halloysite and polymer matrix, as well as the good dispersion of reactive halloysite in the polymer matrix, improved the mechanical property of the nanocomposites.

## 4. Conclusions

In this study, PMMA/reactive halloysite nanocomposites were prepared successfully and the effect of the reactive halloysite on the property of PMMA nanocomposites was investigated systematically. The reactive halloysite had good dispersion in the polymer matrix. The temperature at 5 wt % loss of PMMA-TH-3% increased by 19.4% compared to pure PMMA. The glass transition temperature of PMMA-TH-3% increased to 130.0 °C from 111.6 °C. The tensile strength and Young’ modulus of PMMA-TH-3% were up to 42.1 MPa and 2.34 GPa, respectively, increased by 31.1% and 30.7%, respectively, compared to pure PMMA. The improved properties were strongly connected to the dispersion of fillers in the polymer and their compatibility. The study indicates promising prospects in the preparation of new structural and functional nanocomposites which are suitable for industrial applications.

## Figures and Tables

**Table 1:** Average molecular weights and polydispersity of the molecular weight distribution of PMMA and PMMA/reactive halloysite nanocomposite.

Samples	*M*_w_	*M*_n_	*M*_w_/*M*_n_
PMMA	585,850	230,915	2.54
PMMA-H-3%	593,488	320,979	1.85
PMMA-TH-3%	665,752	457,961	1.45

**Table 2:** The decomposition temperatures of the samples at 5 wt % and 10 wt % loss.

Samples	Temperature (°C) 5 wt % Loss	Temperature (°C) 10 wt % Loss
PMMA	237	305
PMMA-H-3%	261	301
PMMA-TH-3%	283	330

**Table 3:** The mechanical properties of PMMA/reactive halloysite nanocomposite films.

Samples	Tensile Strength (MPa)	Elongation at Break (%)	Young’s Modulus (GPa)
PMMA	32.1 ± 0.4	5.1 ± 0.4	1.79 ± 0. 08
PMMA-TH-1%	34.0 ± 0.2	2.2 ± 0.1	2.12 ± 0. 09
PMMA-TH-3%	42.1 ± 0.3	2.5 ± 0.2	2.34 ± 0. 08
PMMA-H-3%	33.0 ± 0.2	2.2 ± 0.1	2.07 ± 0. 06
PMMA-KH-3%	34.6 ± 0.3	2.2 ± 0.2	2.26 ± 0. 08
PMMA-TH-5%	34.7 ± 0.3	2.3 ± 0.2	1.83 ± 0. 06
